# Incorporation characteristics of exogenous ^15^N-labeled thymidine, deoxyadenosine, deoxyguanosine and deoxycytidine into bacterial DNA

**DOI:** 10.1371/journal.pone.0229740

**Published:** 2020-02-27

**Authors:** Kenji Tsuchiya, Tomoharu Sano, Noriko Tomioka, Ayato Kohzu, Kazuhiro Komatsu, Ryuichiro Shinohara, Shinji Shimode, Tatsuki Toda, Akio Imai

**Affiliations:** 1 Faculty of Science and Engineering, Soka University, Tangi, Hachioji, Tokyo, Japan; 2 Center for Regional Environmental Research, National Institute for Environmental Studies, Onogawa, Tsukuba, Ibaraki, Japan; 3 Center for Environmental Measurement and Analysis, National Institute for Environmental Studies, Onogawa, Tsukuba, Ibaraki, Japan; 4 Graduate School of Environment and Information Science, Yokohama National University, Tokiwadai, Hodogaya, Yokohama, Kanagawa, Japan; University of Hyogo, JAPAN

## Abstract

Bacterial production has been often estimated from DNA synthesis rates by using tritium-labeled thymidine. Some bacteria species cannot incorporate extracellular thymidine into their DNA, suggesting their biomass production might be overlooked when using the conventional method. In the present study, to evaluate appropriateness of deoxyribonucleosides for evaluating bacterial production of natural bacterial communities from the viewpoint of DNA synthesis, incorporation rates of four deoxyribonucleosides (thymidine, deoxyadenosine, deoxyguanosine and deoxycytidine) labeled by nitrogen stable isotope (^15^N) into bacterial DNA were examined in both ocean (Sagami Bay) and freshwater (Lake Kasumigaura) ecosystems in July 2015 and January 2016. In most stations in Sagami Bay and Lake Kasumigaura, we found that incorporation rates of deoxyguanosine were the highest among those of the four deoxyribonucleosides, and the incorporation rate of deoxyguanosine was approximately 2.5 times higher than that of thymidine. Whereas, incorporation rates of deoxyadenosine and deoxycytidine were 0.9 and 0.2 times higher than that of thymidine. These results clearly suggest that the numbers of bacterial species which can incorporate exogenous deoxyguanosine into their DNA are relatively greater as compared to the other deoxyribonucleosides, and measurement of bacterial production using deoxyguanosine more likely reflects larger numbers of bacterial species productions.

## Introduction

In aquatic ecosystems, bacteria play key roles to drive the microbial loop using dissolved/particulate organic matter as their substrates, and to transfer energy and materials to higher trophic levels as well as conventional grazing food chains [1]. To estimate relative importance of externally produced (allochthonous) and internally produced (autochthonous; i.e. algal primary production) organic matter loadings for supporting the ecosystems, measuring bacterial production is necessary since bacterial productions are often higher than primary productions [2]. The conventional methods of measuring bacterial production in seawater and freshwater ecosystems are tritium-thymidine method (^3^H-dT method; [3, 4]) and tritium-leucine method (^3^H-Leu method; [5]). In this method, bacteria incorporate radioisotope (tritium; ^3^H) labeled thymidine or leucine into their DNA or protein, respectively, and the incorporation rate is measured to estimate bacterial production. Although this method provides methodological simplicity and high sensitivity, the use of radioisotopes is often restricted by regulation, particularly in field settings. Tsuchiya et al. [6] developed a non-radioactive method to quantify directly nucleosides incorporated into bacterial DNA in fresh- and seawater ecosystems, and ^15^N_5_-2’-deoxyadenosine (^15^N-dA) labeled by nitrogen stable isotopes (SI) is used instead of ^3^H-dT (^15^N-dA method). In the procedure of ^15^N-dA method, bacterial DNA is extracted after incubation, the extracted DNA is enzymatically hydrolyzed to nucleosides, and then the incorporated ^15^N-dA is directly quantified by using liquid chromatography mass spectrometry (LC-MS). The direct quantification of the incorporated nucleosides can obtain pure DNA synthesis rates, which should solve a problem of radio-isotopic non-specific labeling in ^3^H-dT method resulting in overestimation of DNA synthesis rate [7].

In natural bacterial communities, some bacterial populations cannot incorporate extracellular dT into their DNA, and especially dominant freshwater bacterial groups such as *Betaproteobacteria* hardly take up dT [8, 9]. The reason for this exclusion is considered lack of thymidine kinase which phosphorylates dT to dTMP [10] and thymidine transport systems [11], and preference for *de novo* synthesis pathway rather than salvage usage [12]. Thus, bacterial production measured by using dT reflects not the production of whole but that of some bacterial populations. In the conventional method, only dT has been used for measuring bacterial production based on DNA synthesis rate although there are other deoxyribonucleosides; deoxyadenosine (dA), deoxyguanosine (dG) and deoxycytidine (dC). This is because dT is not a component of RNA, thus dT has been considered not to be incorporated into RNA. The ^15^N-dA method can quantify directly incorporated stable isotope labeled deoxyribonucleosides into DNA [6] though some labeled deoxyribonucleosides are incorporated into RNA and other macromolecules such as protein. If the incorporation rates of other deoxyribonucleosides into DNA are higher than that of dT, the number of bacterial species which can incorporate these deoxyribonucleosides into their DNA can be larger, expecting higher accuracy of evaluating bacterial production of whole bacterial populations in aquatic ecosystems.

Therefore, we evaluated incorporation characteristics of all four deoxyribonucleosides (dT, dA, dG and dC) in freshwater and seawater ecosystems by using the ^15^N-dA method [6] to examine the appropriateness of deoxyribonucleosides for measuring bacterial production. In the present study, we used ^15^N-labeled deoxyribonucleosides; ^15^N_2_-thymidine, ^15^N_5_-deoxyadenosine, ^15^N_5_-deoxyguanosine and ^15^N_3_-deoxycytidine.

## Materials and methods

### Sampling

Bacterial samples were collected from Sagami Bay and Lake Kasumigaura, Japan, in summer (July 2015) and winter (January 2016). In Sagami Bay, seawater samples were collected at Manazuru Port, inshore station (St. A; 5 m depth; 35° 08.9’ N, 139° 09.1’ E), and the offshore-shelf station (St. M; 120 m depth; 35° 09.0’ N, 139° 10.5’ E). The sampling at St. M was conducted aboard the R.V. Tachibana of the Manazuru Marine Center for Environmental Research and Education (MMCER), Yokohama National University. Surface seawater was collected with a bucket, and seawater from 30 and 100 m depths by 5 L Niskin bottles. Seawater at 30 and 100 m depths were collected only in January 2016. Collected water samples were pre-screened through a 180 μm nylon mesh to remove large zooplankton and debris, and were immediately brought back to the field laboratory of MMCER. Lake Kasumigaura basin is smooth and shallow, with a mean depth of 4.0 m, and a maximum depth of 7.3 m [13]. Lake water samples were collected at Takahama bay station (St. 1; 36° 08.952’ N, 140° 19.492’ E), Tsuchiura bay station (St. 7; 36° 03.902’ N, 140° 13.993’ E), the lake center station (St. 9; 36° 02.142’ N, 140° 24.222’ E) and the near outlet station (St. 12; 35° 58.593’ N, 140° 28.332’ E) with a 2-m column sampler. The sampling was conducted aboard the R.V. NIES’94 of National Institute for Environmental Studies (NIES). The samples were immediately brought back to our laboratory.

### Sample preparation (incubation, DNA extraction and DNA enzymatic hydrolysis)

Ten to fifty mL of seawater and lake water were poured to acid-cleaned 60 mL polyethylene bottles, and each of ^15^N_2_-thymidine (^15^N-dT; NLM-3901-25, Cambridge Isotope Laboratories), ^15^N_5_-deoxyadenosine (^15^N-dA; NLM-3895-25, Cambridge Isotope Laboratories), ^15^N_5_-deoxyguanosine (^15^N-dG; NLM-3899-CA-25, Cambridge Isotope Laboratories) and ^15^N_3_-deoxycytidine (^15^N-dC; NLM-3897-25, Cambridge Isotope Laboratories) was added to each bottle (final concentration 50 nM). This final concentration was determined in the previous study [6]. The water samples were incubated under dark condition at in situ temperature for 5 to 24 hours. After the incubation, the water samples were filtrated onto a 0.2 μm PTFE membrane filter (Omnipore, Millipore), and the filter was stored at −80°C until further analysis. DNA extraction was conducted by using Extrap Soil DNA Kit Plus ver.2 (Nippon Steel & Sumikin Eco-Tech Corporation) with which bacterial cells were broken by glass beads using Fast Prep FP120 (Speed 6.0, Time 40 second; MP Biomedical). Extractions were performed in accordance with the manufacturer’s protocols. The DNA extraction efficiency was considered as 100% [14]. After the DNA extraction, the DNA sample was enzymatically hydrolyzed to deoxyribonucleosides according to Nohara et al. [15] with minor modifications. The extracted DNA sample was denatured by heating at 98 °C for 10 min, and the sample was then quenched rapidly on ice. The sample was incubated with two units of nuclease P1 (Wako) at 60 °C for 2 h in 10 mM ammonium acetate, with 0.002 units of phosphodiesterase I (Worthington, Lakewood, NJ, USA) at 37 °C for 2 h in 100 mM ammonium bicarbonate, and then with 0.5 units of alkaline phosphatase (Promega, Fitchburg, WI, USA) at 37 °C for 1 h.

### LC-MS/MS analysis

After the enzymatic hydrolysis, the amount of ^15^N-dT, ^15^N-dA, ^15^N-dG and ^15^N-dC incorporated during the incubation were analyzed by LC-MS/MS using ^13^C_10_^15^N_2_-thymidine (CNLM-3902-PK, Cambridge Isotope Laboratories Inc.), ^13^C_10_^15^N_5_-deoxyadenosine (CNLM-3896-CA, Cambridge Isotope Laboratories Inc.), ^13^C_10_^15^N_5_-deoxyguanosine (CNLM-3900-PK, Cambridge Isotope Laboratories Inc.) and ^13^C_9_^15^N_3_-deoxycytidine (CNLM-3898-LAS-5, Cambridge Isotope Laboratories Inc.) as a surrogate, respectively. LC-MS/MS analysis was performed by LCMS-8040 liquid chromatography mass spectrometer (Shimadzu) with Nexera X2 liquid chromatograph system (Shimadzu). The analysis conditions were described as follows; column: Atlantis dC18, 5 μm, 2.1 × 150 mm (Waters); retention gap column: Atlantis T3, 3 μm, 2.1 × 150 mm (Waters); solvent A: 10 mM ammonium acetate aqueous solution; solvent B: methanol; column oven temperature: 40 °C; flow rate: 0.2 mL min^-1^. The deoxyribonucleosides were separated by 2% methanol for 5 min, 8% methanol for 4 min, a linear gradient condition from 8% to 40% methanol for 6 min, and then 40% methanol until 18 min. The dC peak was eluted around 4.5 min. The dC, ^15^N-dC and ^13^C_9_^15^N_3_-dC were detected with positive MRM mode using *m/z* 228.00> 112.05, 231.00 > 115.10, and 240.00 > 119.05, respectively (S1 Fig). The dG peak was eluted around 11 min. The dG, ^15^N-dG and ^13^C_10_^15^N_5_-dG were detected with positive MRM mode using *m/z* 268.00 > 152.10, 273.00 > 157.05, and 283.00 > 162.00, respectively. The dT peak was eluted around 12 min. The dT, ^15^N-dT and ^13^C_10_^15^N_2_-dT were detected with positive MRM mode using *m/z* 243.00 > 127.05, 245.00 > 129.05, and 255.20 > 134.10, respectively. The dA peak was eluted around 16 min. The dA, ^15^N-dA, and ^13^C_10_^15^N_5_ -dA were detected with positive MRM mode using *m/z* 252.00 > 136.05, 257.00 > 141.05, and 267.00 > 146.10, respectively. The hydrolyzed DNA solution (15 μL) was mixed with ^13^C_10_^15^N_5_-dA, ^13^C_10_^15^N_2_-dT, ^13^C_10_^15^N_5_-dG and ^13^C_9_^15^N_3_-dC (15 μL of 4 ng mL^-1^ solution), before a 10 μL sample was injected into LC-MS/MS.

## Results

**Fig 1:**
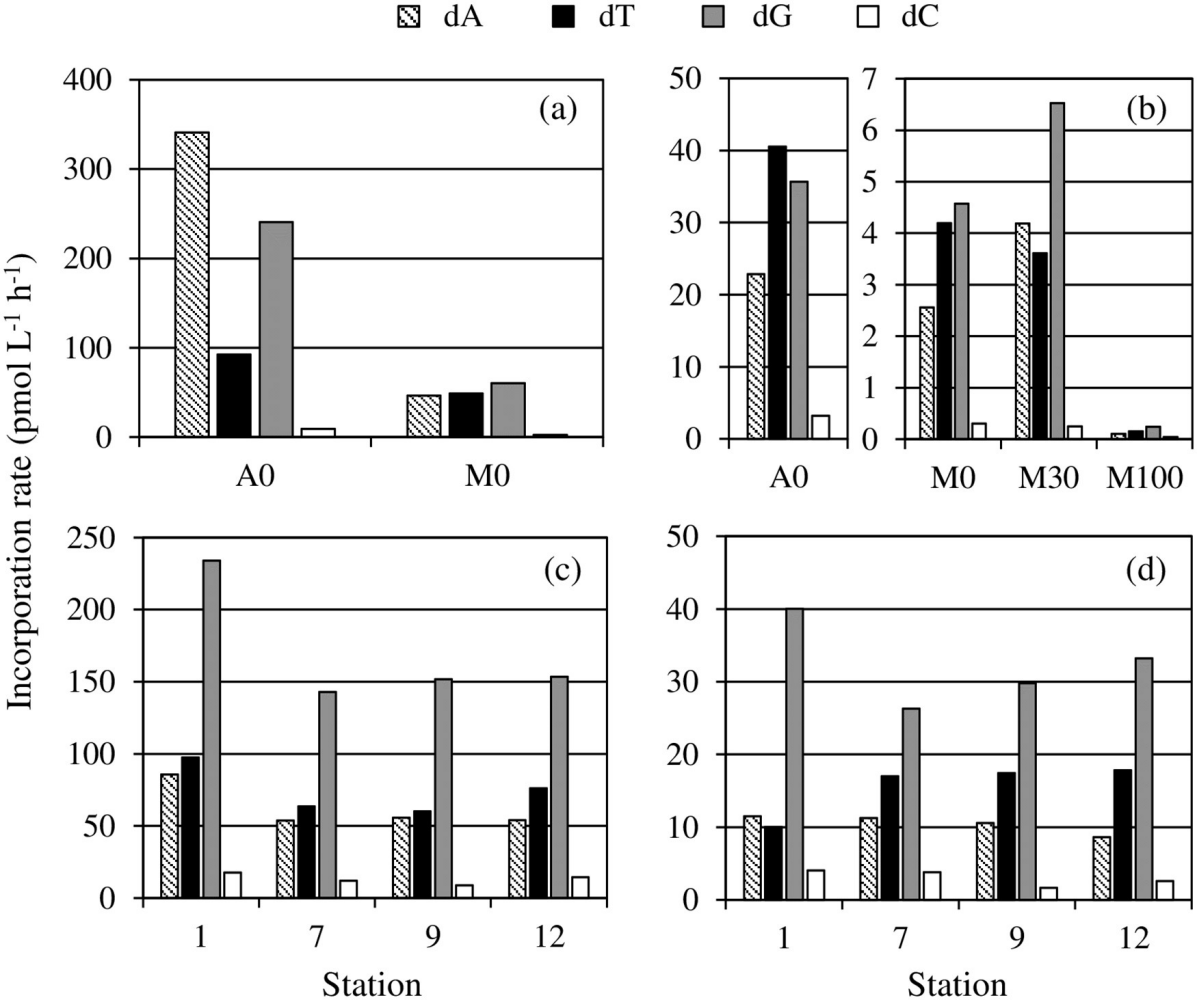
Incorporation rate of each deoxyribonucleoside. (a) Sagami Bay in July 2015. (b) Sagami Bay in January 2016. (c) Lake Kasumigaura in July 2015. (d) Lake Kasumigaura in January 2016.

At St. A in Sagami Bay, the highest incorporation rates of deoxyribonucleosides were found for dA in July and dT in January, and the second was dG in both of July and January (Fig 1). On the other hand, dG showed the highest incorporation rate at all depths of St. M in both of July and January. Incorporation rates of dC were the lowest among the four deoxyribonucleosides at all stations in any seasons. In Lake Kasumigaura, dG showed the highest incorporation rates at all stations in both of July and January, and dT was almost the second fastest deoxyribonucleosides. As well as Sagami Bay, incorporation rates of dC were the slowest among the four deoxyribonucleosides in Lake Kasumigaura.

**Fig 2:**
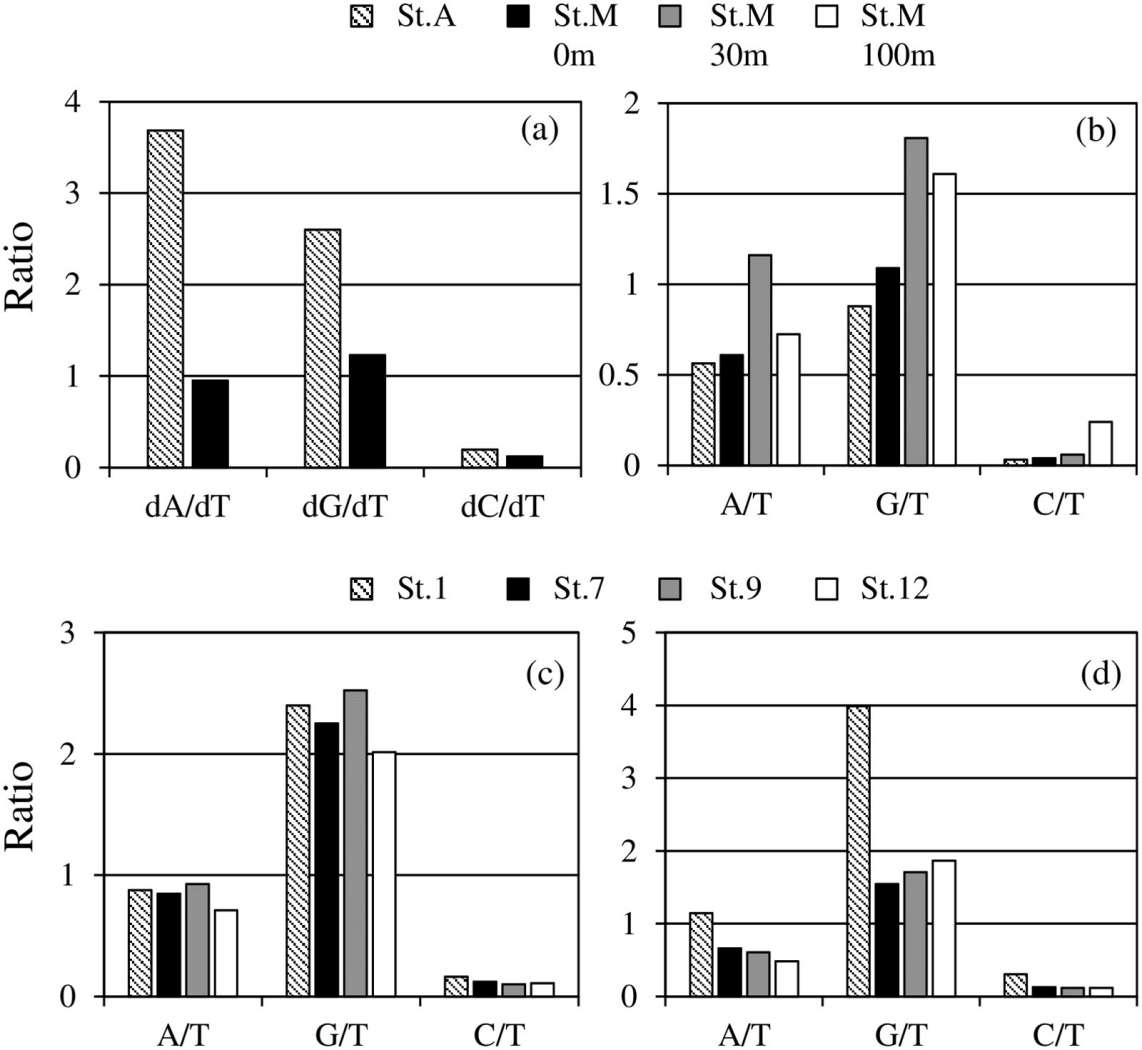
Ratios of incorporation rates of each deoxyribonucleoside. (a) Sagami Bay in July 2015. (b) Sagami Bay in January 2016. (c) Lake Kasumigaura in July 2015. (d) Lake Kasumigaura in January 2016.

In Sagami Bay in July, dA/dT and dG/dT ratios were relatively high (>2) at St. A, whereas the ratios were around 1 at the surface of St. M (Fig 2a). The dC/dT ratios at both stations showed low values. In January, the dG/dT ratio at all depths of St. M was greater than 1 (Fig 2b), whereas the dG/dT ratio at St. A was 0.880. The dA/dT ratios were less than 1, showing 0.564–0.724 except for 30 m depth of St. M. The dC/dT ratios of all stations were 0.0319–0.240, suggesting dC showed the lowest incorporation rates among the four deoxyribonucleosides in Sagami Bay. In Lake Kasumigaura, the ratios showed similar values at all stations, and the dG/dT ratio was the highest, 2.02–2.40 in July (Fig 2c). The dC/dT ratios in July were the lowest, 0.100–0.163, as is the case for Sagami Bay. In January, there observed difference between St. 1 and other stations in the incorporation ratios, and the dG/dT ratio at St. 1 was 3.99, the highest among the stations (Fig 2d). The dA/dT ratio at St. 1 also showed relatively high values compared to those of the other stations. dC/dT ratios at all stations were low, 0.117–0.305. To examine whether the ratios of each deoxyribonucleoside incorporation rate was affected by the DNA GC content or not, the relationships between the GC content (36.9–47.7%), and the dA/dT, dG/dT and dC/dT ratios in both of Sagami Bay and Lake Kasumigaura were examined. However, there were no significant relationships between the ratios of dA/dT, dG/dT and dC/dT, and GC contents (figure not shown).

**Fig 3:**
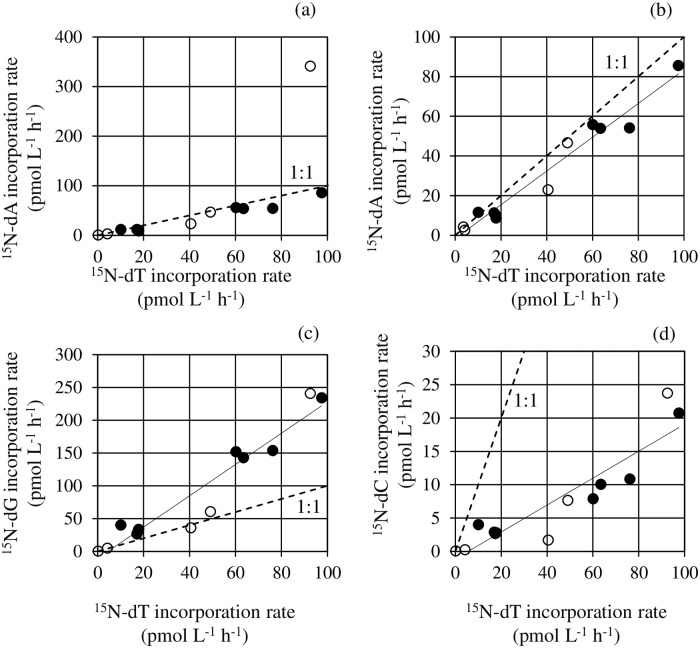
Relationships between incorporation rates of deoxyribonucleosides. (a) dA and dT. (b) dA and dT excluding an apparent outliner shown in (a). (c) dG and dT. (d) dC and dT. The regression lines obtained by model II linear regression were (b) [dA] = 0.87×[dT]– 2.3, (*n* = 13, *r*^*2*^ = 0.96, *p* < 0.001), (c) [dG] = 2.5×[dT]– 15 (*n* = 14, *r*^*2*^ = 0.92, *p* < 0.001) and (d) [dC] = 0.22×[dT]– 1.8 (*n* = 14, *r*^*2*^ = 0.85, *p* < 0.001). White and black circles represent Sagami Bay and Lake Kasumigaura, respectively. The dotted line represents 1:1 incorporation.

The relationships between the incorporation rate of dT (x-axis), and those of dA, dG and dC (y-axis) were shown in Fig 3. An apparent outliner shown in Fig 3a (the incorporation rate of dA at St. A in July) was excluded from the regression analysis since the outliner was beyond the 95% prediction interval of the regression line of Fig 3b (95% interval, 64 to 92 pmol L^-1^ h^-1^ when dT = 93 pmol L^-1^ h^-1^). All these relationships were significant, and the linear regression formulae were [dA] = 0.87×[dT]– 2.3, (*n* = 13, *r*^*2*^ = 0.96, *p* < 0.001; Fig 3b), [dG] = 2.5×[dT]– 15 (*n* = 14, *r*^*2*^ = 0.92, *p* < 0.001; Fig 3c), and [dC] = 0.22×[dT]– 1.8 (*n* = 14, *r*^*2*^ = 0.85, *p* < 0.001; Fig 3d). The 97.5% confidence intervals of these regression slopes were shown in Table 1. The slope between dA and dT was 0.87±0.11, meaning almost the same incorporation rates between dA and dT. The slope between dG and dT was 2.49±0.44, suggesting approximately 2.5 times higher of dG incorporation rates compared to that of dT. Whereas, the slope between dC and dT was 0.22±0.05, and it was made clear that dC incorporation rate was just approximately one fourth compared to that of dT.

**Table 1:** Relative incorporation rates of ^15^N-dA and ^15^N-dT, ^15^N-dG against that of ^15^N-dT. ± represents 97.5% confidence interval of the relative incorporation rates.

dA/dT	dG/dT	dC/dT
0.87±0.11	2.49±0.44	0.22±0.05

**Fig 4:**
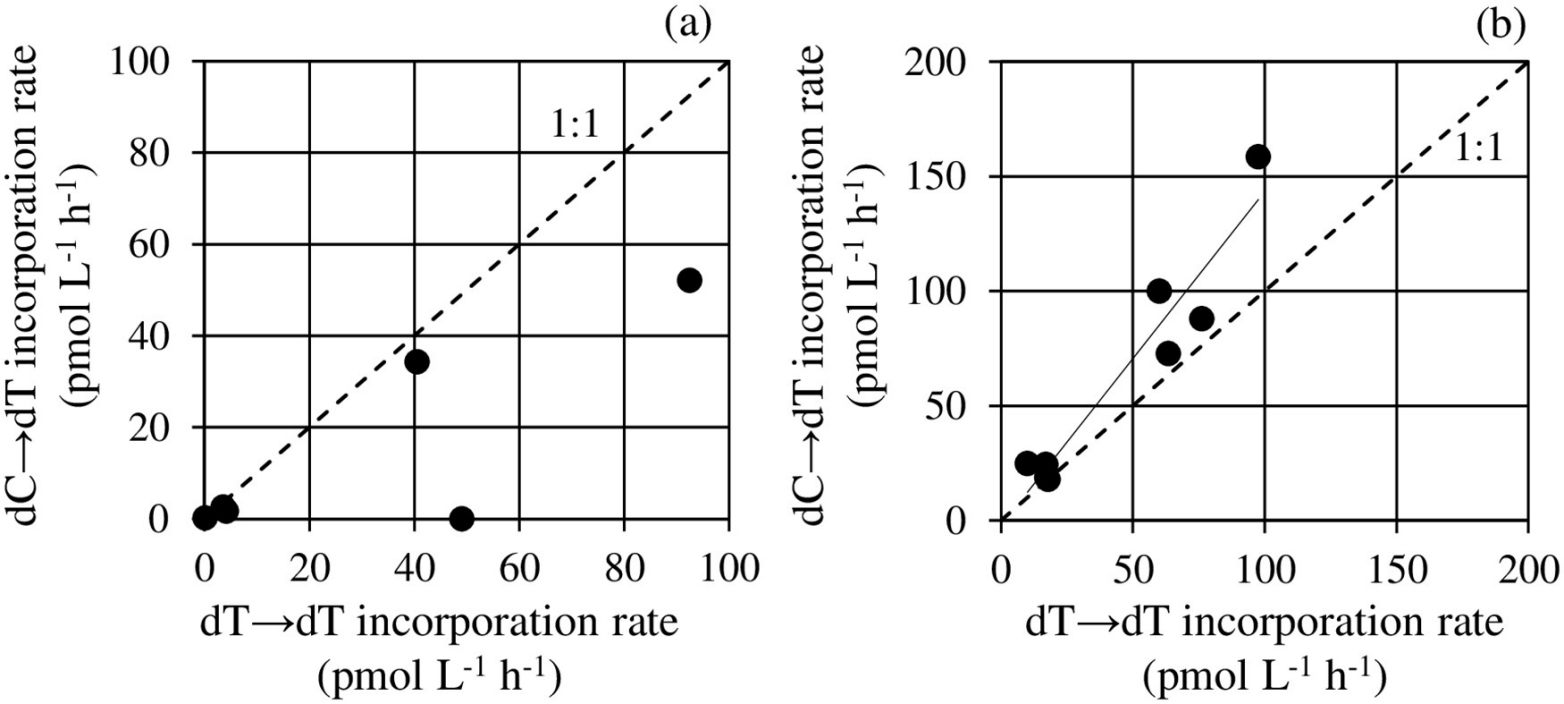
Relationships of dT incorporation rates between samples incubated with ^15^N-dT and ^15^N-dC. (a) Sagami Bay. (b) Lake Kasumigaura. The regression line in Lake Kasumigaura was significant, and the slope of the regression line was more than 1 ([dC→dT] = 1.5 × [dT→dT]– 2.4; *n* = 8, *r*^*2*^ = 0.92, *p* < 0.001). The dotted line represents 1:1 incorporation.

We quantified the amount of dT in the samples incubated with ^15^N-dC to examine a potential salvage pathway from dC to dT in bacterial cells. The incorporation rate of dT incubated with ^15^N-dC ranged from values lower than the detection limit to 158 pmol L^-1^ h^-1^ in July, and from 0.167 to 34.3 pmol L^-1^ h^-1^ in January, which were comparable to the dT incorporation rate incubated with ^15^N-dT. The relationships of dT incorporation rates between samples incubated with ^15^N-dT and ^15^N-dC were shown in Fig 4. In Sagami Bay, the dT incorporation rate incubated with ^15^N-dC were lower than that incubated with ^15^N-dT (Fig 4a), whereas in Lake Kasumigaura there found a significant linear relationship between them, and the slope was 1.46, suggesting the dT incorporation rate incubated with ^15^N-dC was higher than that incubated with ^15^N-dT (Fig 4b).

## Discussion

In the present study, the dG incorporation rate was the highest among the four deoxyribonucleosides in both of marine and freshwater ecosystems, and the dG incorporation rate was approximately 2.5 times higher than that of dT. Furthermore, the dG/dT ratio was not significantly correlated to the GC content, indicating the GC content could not explain the highest incorporation rate of dG in natural bacterial communities. These results suggest that bacterial species which were able to incorporate dG into their DNA was larger in number than those of the other deoxyribonucleosides, and use of dG for measuring bacterial production could reflect the production of a larger number of bacterial species compared with the other deoxyribonucleosides.

In freshwater ecosystem, only 15–24% of bacteria were able to take up dT though leucine was taken up by 70–80% of bacteria [9]. Especially only less than 3% of *Betaproteobacteria*, which is a major bacterial group in freshwater ecosystems [16], took up dT, whereas 92% of the cells were able to absorb leucine [9]. In that case, the bacterial production measured by using dT might lead to an underestimation of the production. Measured by using dG, the bacterial production may potentially reflect that due to 37~60% of bacterial cells (15–24%×2.49 = 37–60%; 2.49 was derived from the dG/dT ratio, Table 1).

Tinta et al. [17] reviewed two types of deoxyribonucleoside kinases (dNKs) from tens of available aquatic bacterial genomes; one was thymidine kinase (TK1), which could mainly phosphorylate thymidine, and the other was non-TK1 dNK, which could mainly phosphorylate deoxyribonucleosides other than thymidine. According to their work, except for Class *Bacteroidetes* and *Firmicutes*, most of bacterial species had either TK1-like or non-TK1-like kinases [17]. The results suggest that the combination of dT and dG for measuring bacterial production covers the production of much more bacterial species from the view point of kinases.

When bacteria incorporate extracellular dC into their DNA, deoxycytidine kinase is necessary in the first step. *Lactobacillus acidophilus*, *Lactobacillus leichmanii* and *Bacillus megaterium* have deoxycytidine kinases [18–21]. On the other hand, some bacteria such as *Escherichia coli*, *Salmonella typhimurium* [22] and *Pneumococcus pneumoniae* [23] do not have any deoxycytidine kinases. The latter bacteria cannot incorporate extracellular dC into their DNA, and should depend on intracellular *de novo* synthesis of dC for their DNA synthesis. The present study, showing the lowest dC incorporation rates among the four deoxyribonucleosides, suggests that major bacterial groups are devoid of deoxycytidine kinases, and reveals the dominance of *de novo* synthesis of dC for DNA synthesis in natural bacterial communities.

**Fig 5:**
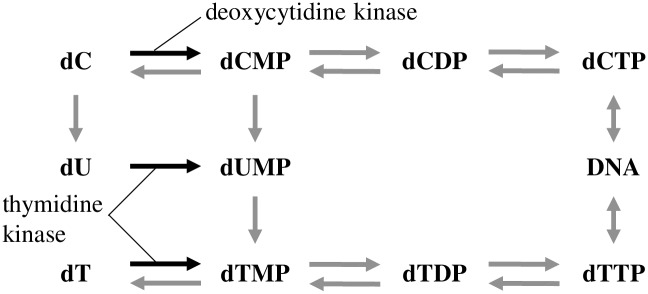
Metabolic pathway of pyrimidine-base nucleosides and nucleotides. dC; deoxycytidine, dCMP; deoxycytidine monophosphate, dCDP; deoxycytidine diphosphate, dCTP; deoxycytidine triphosphate, dU; deoxyuridine, dUMP; deoxyuridine monophosphate, dT; thymidine, dTMP; thymidine monophosphate, dTDP; thymidine diphosphate, dTTP; thymidine triphosphate. dC is phosphorylated by deoxycytidine kinase, and dU and dT are phosphorylated by thymidine kinase.

Salvage pathway of incorporated dC to dT (accurately thymidine monophosphate; dTMP) was demonstrated in a single bacterial strain [24]. The present study confirmed the occurrence or presence of salvage usage even in natural bacterial communities (Fig 4). However, the extent of the salvage usage was different between Sagami Bay and Lake Kasumigaura; in Sagami Bay, the dT incorporation rate incubated with ^15^N-dC was lower than that incubated with ^15^N-dT, whereas in Lake Kasumigaura the dT incorporation rate incubated with ^15^N-dC was higher than that incubated with ^15^N-dT (Fig 4). In freshwater ecosystems, one of the dominant bacterial groups is *Betaproteobacteria*, which have no thymidine kinases [17], suggesting they cannot incorporate extracellular dT into their DNA. Meanwhile, incorporated dC can be used to synthesize their DNA as thymine (Fig 5). The plausible metabolic pathway is the following; incorporated dC is converted into deoxycytidine monophosphate (dCMP) by deoxycytidine kinases, deoxyuridine monophosphate (dUMP) by dCMP deaminase, thymidine monophosphate (dTMP) by thymidylate synthase [25], and finally DNA as thymine. Thus freshwater bacterial communities should incorporate extracellular dC into their DNA as thymine indirectly by using the potential metabolic pathway initiated by deoxycytidine kinases. Whereas, in marine ecosystems, *Alphaproteobacteria* are one of the dominant groups [26] and most of the members have thymidine kinases [17]. Therefore, marine bacterial communities consumed extracellular dT to incorporate into their DNA as thymine directly, and the necessity to use extracellular dC for their DNA synthesis as thymine should be lower than that of freshwater bacterial communities.

In the present study, the salvage usages were detected in the other deoxyribonucleosides; ^15^N-dA→^15^N-dG, ^15^N-dG→^15^N-dA, ^15^N-dT→^15^N-dC and ^15^N-dC→^15^N-dT (S2 Fig). The ratio of ^15^N-dG to ^15^N-dA incorporation rate incubated with ^15^N-dA (dA→dG/dA→dA) was 0.20 ± 0.17 in average, suggesting at least 20% of ^15^N-dA taken into bacterial cells were converted to dG and incorporated into DNA as dG. Whereas, The ratio of ^15^N-dA to ^15^N-dG incorporation rate incubated with ^15^N-dG (dG→dA/dG→dG) was 0.035 ± 0.025 in average, which was significantly lower than dA→dG/dA→dA ratio (*p* < 0.0001, *Student’s* t-test). The plausible explanations were that the biochemical metabolic pathway of dG→dA required higher energy or cost than that of dA→dG, or *de novo* synthesis of dG was higher in cost compared to dA as discussed below. The relative rate of dC→dT/dC→dC was quite high (9.9 ± 6.3), whereas there was scarcely any salvage usage of dT→dC (dT→dC/dC→dC ratio = 0.0015 ± 0.0031, *n* = 3; S2 Fig). There were two plausible explanations; 1) bacteria did not use dT as the source of dC for DNA synthesis, leading to a negligible amount of the metabolic salvage pathway of dT→dT, or 2) LC-MS/MS could not detect ^15^N-dC mass spectrometrically since the number of ^15^N in the nucleosides were different (^15^N_2_-dT and ^15^N_3_-dC). In future work, we need to use other stable isotope-labeled dT to clarify whether dT can be salvaged into dC in bacterial biochemical metabolism.

There are two plausible reasons why dG incorporation rate was the highest among the four deoxyribonucleosides; 1) there is a larger number of bacterial species which have enzymes phosphorylating dG and 2) *de novo* synthesis of dG might be higher in cost compared to that of the other deoxyribonucleosides, and salvage pathway of dG might be much reasonable for bacteria. As to 1), aquatic bacteria genomes should be examined for the presence of enzymes to phosphorylate dG as well as the work of [17] to test the hypothesis. As to 2), the cost of *de novo* synthesis and salvage usage should be varied depending on bacterial species, and this is beyond our primary purpose of this study. However, guanine nucleotide-binding proteins or GTPases constitute a mechanism for controlling multiple biochemical pathways such as cell division and initiation of protein synthesis within the cellular environment [27–29]. Since guanine tends to be used in cellular physiological processes shown above, bacteria may incorporate extracellular dG into their DNA more diligently rather than the other deoxyribonucleosides.

The LC-MS/MS measurement can acquire the fraction of ^15^N-labeled to total deoxyribonucleoside (dN; ratio of ^15^N-dN/total dN shown in S3 Fig) using mass spectral data (S1 Fig). Although we did not measure time-course incorporation rates of each ^15^N-dN in the present study, overall microbial turnover rate can be estimated by tracking time-course change of the ratio of ^15^N-dN/total dN itself as following analysis. Isotopic enrichment resulting from unbiased clonal growth in the presence of an isotopic label can be described by the following equation:

$$F(t)=F_{L}\times \;(1\;-\;e^{-\mu t})\;+\;F_{0}\times \;e^{-\mu t}$$ (1)

where *F*(t) is the isotopic enrichment (%) at time t (h), *F*_L_ is the maximum isotopic composition that can be provided by the isotopic label, *F*_0_ is the isotopic composition of native biomass and μ is the specific growth rate (h^−1^) [30]. In the present study, it is safe to assume that *F*_0_ = 0% since natural abundance of ^15^N-dA in native biomass is almost zero (6.39 × 10^−13^; [6]), simplifying this equation to:

$$F(t)=F_{L}\times \;(1\;-\;e^{-\mu t})$$ (2)

The time course data of ^15^N-labeled proportion (%) vs incubation time derived from Tsuchiya et al. [6] was analyzed to estimate *F*_L_ and μ according to Eq 2 (S4 Fig). We assumed that 1) cells would use the exogenous nucleosides preferentially (or at the very least at some fixed proportion) to synthesize new DNA thus leading to the characteristic 1 − *e*^−μt^ initial uptake pattern, and 2) this exogenous source became depleted at some point (the maximum in the enrichment curves in S4 Fig) that depended on the amount provided originally, at which point the microbial community reverted back to *de novo* biosynthesis and started diluting the ^15^N labeled pool of nucleosides from the DNA again. The estimated μ ranged from 0.0496 to 0.373 h^−1^, corresponding to generation time (= ln2 / μ) of 1.86 to 14.0 h (S1 Table). The analysis provides μ and generation time purely from the mass-spectrometric data without the need for any other cell number estimates and some of the other issues associated with converting the incorporation rates themselves into generation time estimates. We should verify the consistency of the parameters estimated by this analysis in all deoxyribonucleosides for the further development of measuring bacterial productivity.

## Supporting information

S1 Fig Mass-spectrometric chromatograph of each deoxyribonucleoside. The mass spectral chromatograph of each deoxyribonucleoside was obtained from the sample incubated with the targeted ^15^N-deoxyribonucleoside.
(PDF)

S2 Fig Salvage usage of incorporated deoxyribonucleosides. (a) The ratio of ^15^N-dG to ^15^N-dA incorporation rate incubated with ^15^N-dA (dA→dG/dA→dA). (b) the ratio of ^15^N-dA to ^15^N-dG incorporation rate incubated with ^15^N-dG (dG→dA/dG→dG). (c) the ratio of ^15^N-dT to ^15^N-dC incorporation rate incubated with ^15^N-dT (dT→dC/dT→dT). (d) the ratio of ^15^N-dC to ^15^N-dT incorporation rate incubated with ^15^N-dC (dC→dT/dC→dC).
(PDF)

S3 Fig Fractions of ^15^N-labeled to total deoxyribonucleoside (dN; ^15^N-dN/total dN). (a) Sagami Bay in July 2015. (b) Sagami Bay in January 2016. (c) Lake Kasumigaura in July 2015. (d) Lake Kasumigaura in January 2016.
(PDF)

S4 Fig Time course of percent ^15^N labeled vs incubation time. The exponential curves (*F*(*t*) = *F*_L_ × (1 − *e*^−μt^)) were fitted by non-linear least squares regression. Data from Tsuchiya et al. 2015, Fig 4.
(PDF)

S1 Table Parameters estimated by the exponential curves (*F*(*t*) = *F*_L_ × (1 − *e*^−μt^)) derived from S4 Fig. Generation times (gen time; h^−1^) were calculated by gen time = ln(2) / μ.
(PDF)
