# Supplementary material for: Incorporation characteristics of exogenous 15N-labeled thymidine, deoxyadenosine, deoxyguanosine and deoxycytidine into bacterial DNA
Source: PLoS One. 2020 Feb 27;15(2):e0229740. doi: 10.1371/journal.pone.0229740 (PMC7046229; doi:10.1371/journal.pone.0229740)
Supplement: S1 Fig — The mass spectral chromatograph of each deoxyribonucleoside was obtained from the sample incubated with the targeted 15N-deoxyribonucleoside. (PDF) [file pone.0229740.s001.pdf]

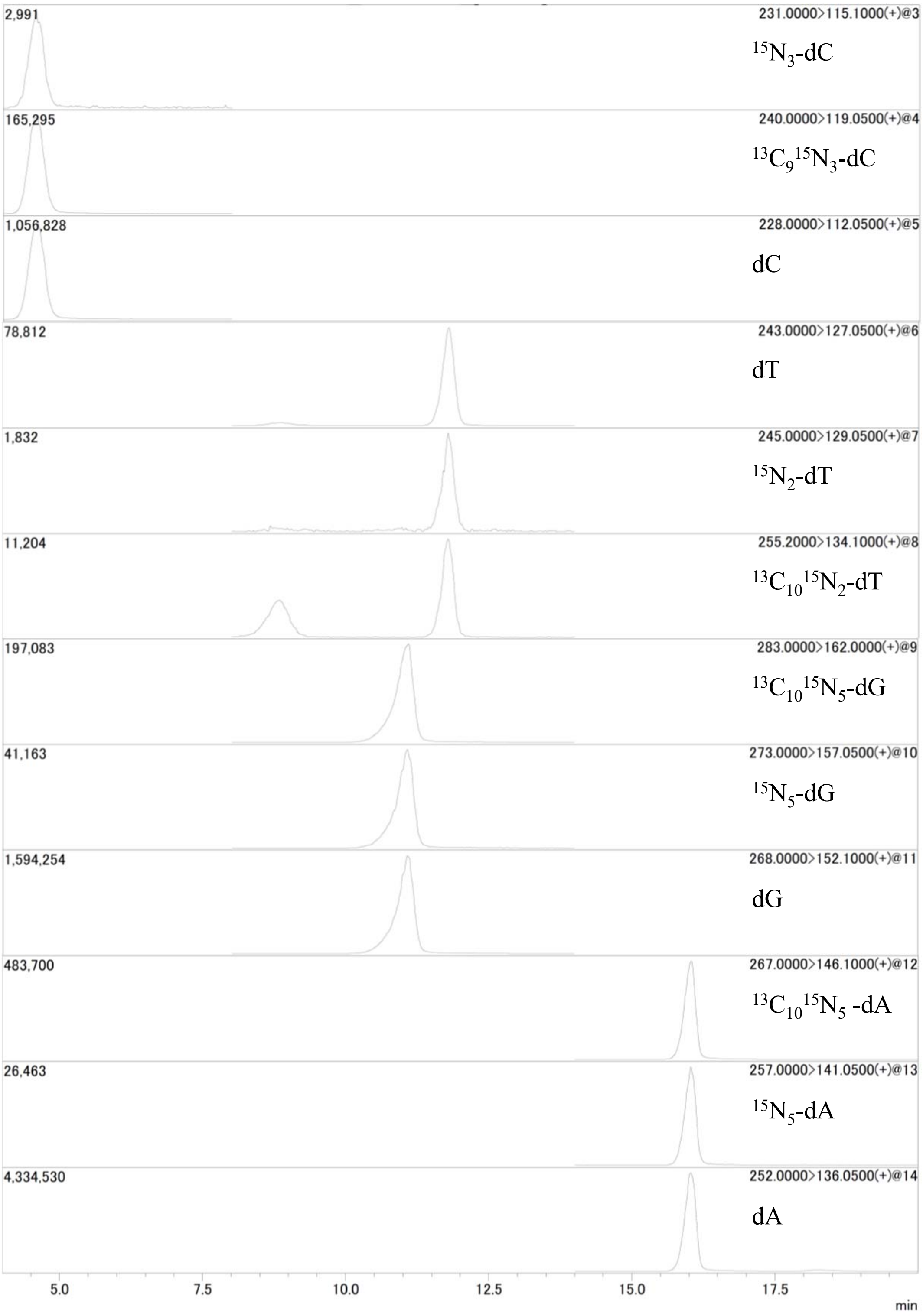

S1 Fig. Mass-spectrometric chromatograph of each deoxyribonucleoside.

The mass spectral chromatograph of each deoxyribonucleoside was obtained from the sample incubated with the targeted ^{15}N-deoxyribonucleoside.
