# Supplementary material for: Incorporation characteristics of exogenous 15N-labeled thymidine, deoxyadenosine, deoxyguanosine and deoxycytidine into bacterial DNA
Source: PLoS One. 2020 Feb 27;15(2):e0229740. doi: 10.1371/journal.pone.0229740 (PMC7046229; doi:10.1371/journal.pone.0229740)
Supplement: S2 Fig — (a) The ratio of 15N-dG to 15N-dA incorporation rate incubated with 15N-dA (dA→dG/dA→dA). (b) the ratio of 15N-dA to 15N-dG incorporation rate incubated with 15N-dG (dG→dA/dG→dG). (c) the ratio of 15N-dT to 15N-dC incorporation rate incubated with 15N-dT (dT→dC/dT→dT). (d) the ratio of 15N-dC to 15N-dT incorporation rate incubated with 15N-dC (dC→dT/dC→dC). (PDF) [file pone.0229740.s002.pdf]

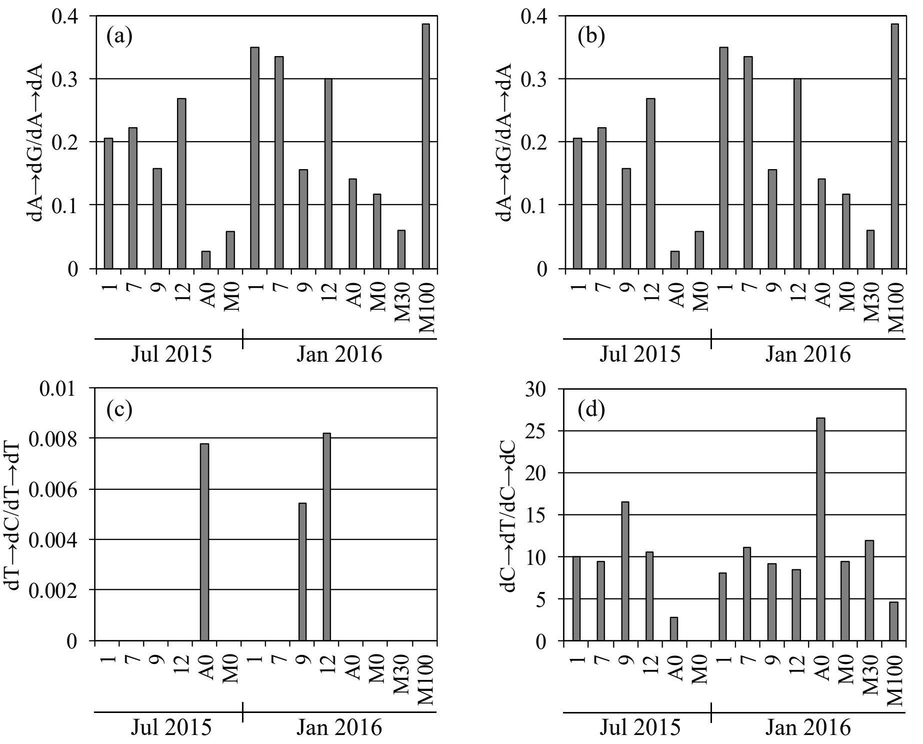

S2 Fig. Salvage usage of incorporated deoxyribonucleosides.

(a) The ratio of ^{15}N-dG to ^{15}N-dA incorporation rate incubated with ^{15}N-dA (dA→dG/dA→dA). (b) the ratio of ^{15}N-dA to ^{15}N-dG incorporation rate incubated with ^{15}N-dG (dG→dA/dG→dG). (c) the ratio of ^{15}N-dT to ^{15}N-dC incorporation rate incubated with ^{15}N-dT (dT→dC/dT→dT). (d) the ratio of ^{15}N-dC to ^{15}N-dT incorporation rate incubated with ^{15}N-dC (dC→dT/dC→dC).
