# Supplementary material for: Incorporation characteristics of exogenous 15N-labeled thymidine, deoxyadenosine, deoxyguanosine and deoxycytidine into bacterial DNA
Source: PLoS One. 2020 Feb 27;15(2):e0229740. doi: 10.1371/journal.pone.0229740 (PMC7046229; doi:10.1371/journal.pone.0229740)
Supplement: S3 Fig — (a) Sagami Bay in July 2015. (b) Sagami Bay in January 2016. (c) Lake Kasumigaura in July 2015. (d) Lake Kasumigaura in January 2016. (PDF) [file pone.0229740.s003.pdf]

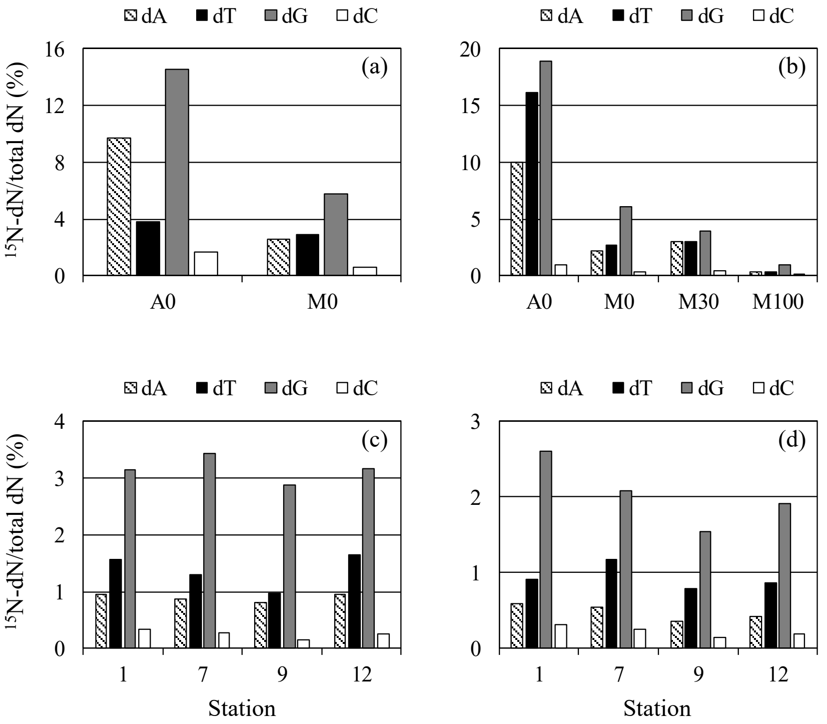

S3 Fig. Fractions of ^{15}N-labeled to total deoxyribonucleoside (dN; ^{15}N-dN/total dN).
(a) Sagami Bay in July 2015. (b) Sagami Bay in January 2016. (c) Lake Kasumigaura in July 2015. (d) Lake Kasumigaura in January 2016.
