# Supplementary material for: Incorporation characteristics of exogenous 15N-labeled thymidine, deoxyadenosine, deoxyguanosine and deoxycytidine into bacterial DNA
Source: PLoS One. 2020 Feb 27;15(2):e0229740. doi: 10.1371/journal.pone.0229740 (PMC7046229; doi:10.1371/journal.pone.0229740)
Supplement: S4 Fig — The exponential curves (F(t) = FL × (1 − e−μt)) were fitted by non-linear least squares regression. Data from Tsuchiya et al. 2015, Fig 4. (PDF) [file pone.0229740.s004.pdf]

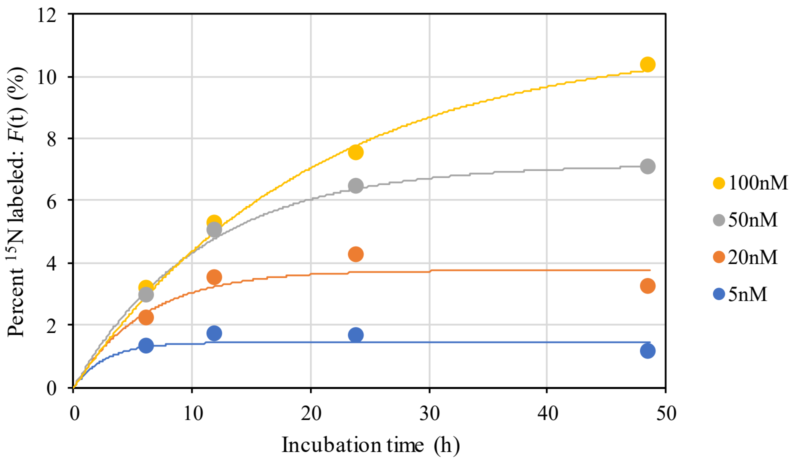

S4 Fig. Time course of percent ^{15}N labeled vs incubation time.

The exponential curves ($F(t) = F_L \times (1 - e^{-\mu t})$) were fitted by non-linear least squares regression. Data from Tsuchiya et al. 2015, Figure 4.
