# Supplementary material for: Incorporation characteristics of exogenous 15N-labeled thymidine, deoxyadenosine, deoxyguanosine and deoxycytidine into bacterial DNA
Source: PLoS One. 2020 Feb 27;15(2):e0229740. doi: 10.1371/journal.pone.0229740 (PMC7046229; doi:10.1371/journal.pone.0229740)
Supplement: S1 Table — Generation times (gen time; h−1) were calculated by gen time = ln(2) / μ. (PDF) [file pone.0229740.s005.pdf]

S1 Table. Parameters estimated by the exponential curves ($F(t) = F_L \times (1 - e^{-\mu t})$) derived from S4 Fig.

Generation times (gen time; h^{-1}) were calculated by gen time = ln(2) / μ.

Conditions	F_L (%)	μ (h^{-1})	gen time (h)
100 nM	11.2	0.0496	14.0
50 nM	7.17	0.0924	7.50
20 nM	3.76	0.165	4.20
5 nM	1.44	0.373	1.86
